# The TAT-RasGAP_317-326_ anti-cancer peptide can kill in a caspase-, apoptosis-, and necroptosis-independent manner

**DOI:** 10.18632/oncotarget.11841

**Published:** 2016-09-02

**Authors:** Mathieu Heulot, Nadja Chevalier, Julien Puyal, Christiane Margue, Sébastien Michel, Stephanie Kreis, Dagmar Kulms, David Barras, Aimable Nahimana, Christian Widmann

**Affiliations:** ^1^ Department of Physiology, University of Lausanne, Lausanne, Switzerland; ^2^ Department of Fundamental Neurosciences, University of Lausanne, Lausanne, Switzerland; ^3^ Signal Transduction Laboratory, Life Sciences Research Unit, University of Luxembourg, Luxembourg, Luxembourg; ^4^ Experimental Dermatology, Department of Dermatology, TU-Dresden, Dresden, Germany; ^5^ Center for Regenerative Therapies, TU-Dresden, Dresden, Germany; ^6^ Bioinformatics Core Facility, Swiss Institute of Bioinformatics, Lausanne, Switzerland; ^7^ Service and Central Laboratory of Hematology, University Hospital of Lausanne, Lausanne, Switzerland

**Keywords:** tumor cell death, non-apoptotic death, cell-permeable peptides, RasGAP

## Abstract

Tumor cell resistance to apoptosis, which is triggered by many anti-tumor therapies, remains a major clinical problem. Therefore, development of more efficient therapies is a priority to improve cancer prognosis. We have previously shown that a cell-permeable peptide derived from the p120 Ras GTPase-activating protein (RasGAP), called TAT-RasGAP_317-326_, bears anti-malignant activities *in vitro* and *in vivo*, such as inhibition of metastatic progression and tumor cell sensitization to cell death induced by various anti-cancer treatments. Recently, we discovered that this RasGAP-derived peptide possesses the ability to directly kill some cancer cells. TAT-RasGAP_317-326_ can cause cell death in a manner that can be either partially caspase-dependent or fully caspase-independent. Indeed, TAT-RasGAP_317-326_-induced toxicity was not or only partially prevented when apoptosis was inhibited. Moreover, blocking other forms of cell death, such as necroptosis, parthanatos, pyroptosis and autophagy did not hamper the killing activity of the peptide. The death induced by TAT-RasGAP_317-326_ can therefore proceed independently from these modes of death. Our finding has potentially interesting clinical relevance because activation of a death pathway that is distinct from apoptosis and necroptosis in tumor cells could lead to the generation of anti-cancer drugs that target pathways not yet considered for cancer treatment.

## INTRODUCTION

Cancer ranks among the leading causes of death worldwide [1]; this makes the development of novel and improved anti-cancer treatment a priority. Current oncological therapeutics aim to activate apoptosis to achieve disease control. However, cancer cells can adapt and become refractory to therapy by mutating and acquiring the ability to resist apoptotic stimuli [2]. The ability to evade apoptosis is one of the hallmarks of cancer [3]. Resistance leads to cancer cell survival and relapse. Consequently, development of treatments able to trigger non-apoptotic forms of death in cancer cells is of prime interest. There are many ways for mammalian cells to die, which could be divided into two main categories: accidental cell death (ACD) or regulated cell death (RCD) [4–6]. ACD is a form of death that is not induced by physiological or pathological insults and that does not involve the signaling machinery of cells. For example, ACD can result from exposure to extreme mechanical or chemical stimuli. In contrast, RCD requires genetically encoded molecular signaling pathways and consequently can be modulated by genetic or pharmacologic interventions. Regulated forms of cell death include the intensively studied apoptosis, necroptosis and autophagy but also less known forms of death such as pyroptosis and parthanatos. These different types of cell death diverge at the level of the morphological changes and biochemical features triggered by death stimuli [4–6].

TAT-RasGAP_317-326_, a cell-permeable peptide derived from the p120 GTPase-activating protein (RasGAP), bears anti-malignant activities, including inhibition of metastatic progression and tumor cell sensitization to cell death induced by anti-cancer therapies [7–12]. This compound, in the initial tumor cell lines tested and at the given doses assessed was not found to affect their viability [7]. By screening additional tumor cells, however, we discovered that some cancer cell lines are directly killed by the RasGAP-derived peptide. The aim of the present study was to investigate which type of death was activated by TAT-RasGAP_317-326_ in the cells that are directly eliminated by the peptide.

## RESULTS

### TAT-RasGAP_317-326_ directly kills a subset of cancer cells

By screening a variety of cancer cell lines for their ability to become more sensitive to genotoxins in the presence of TAT-RasGAP_317-326_, we found 13 tumor cell lines that were directly killed by the peptide (Table 1). Among these, five are B-cell-derived cell lines (Daudi, Namalwa, Raji, Ramos and SKW6.4). To determine if non-transformed B cells were affected by the peptide, purified B cells (CD19^+^ cells) from healthy donors were tested. Supplementary Figure S1 shows that normal B cells were killed by TAT-RasGAP_317-326_ while peripheral blood lymphocytes (PBL) were barely affected. This indicates that the RasGAP-derived peptide can be detrimental to a fraction of immune circulating cells and this will have to be taken into account in case the peptide is therapeutically used.

**Table 1:** Cell lines tested for their sensitivity to TAT-RasGAP_317-326_

	TAT-RasGAP_317-326_ (μM)					
Cell line	10	20	40	60	80	Ref
293T (embryonic kidney)	ND	-	-	ND	+	Unpublished
501Mel (melanoma)	ND	-	-	ND	ND	Unpublished
A375 (melanoma)	-	-	++	++	ND	Unpublished
A673 (Ewing sarcoma)	-	ND	ND	ND	ND	[12]
CCRF-CEM (acute T cell leukemia)	-	ND	ND	ND	ND	[12]
Daudi (Burkitt lymphoma)	-	++	++	ND	ND	Unpublished
EW-11 (Ewing sarcoma)	-	ND	ND	ND	ND	[12]
H1299 (non-small cell lung carcinoma)	ND	-	ND	ND	ND	[46]
HaCat (non-tumor keratinocyte)	ND	-	ND	ND	ND	[7]
HCT116 (colorectal carcinoma)	ND	-	ND	ND	ND	[7]
HeLa (cervical cancer)	-	-	+	ND	++	[7] / Unpublished
H-meso1 (lung mesothelioma)	ND	-	ND	ND	ND	[7]
HUVECC (non-tumor endothelial cells)	ND	-	ND	ND	ND	[7]
IGr37 (melanoma)	ND	-	-	ND	ND	Unpublished
IPC298 (melanoma)	ND	-	-	ND	ND	Unpublished
Jurkat (acute T cell leukemia)	-	-	ND	ND	ND	Unpublished
LAN-1 (neuroblastoma)	-	ND	ND	ND	ND	[12]
Lymphocytes (human PBL)	-	-	+	ND	+	[12] / Unpublished
MCF-7 (breast cancer)	ND	-	ND	ND	ND	[7]
MEF (mouse embryo fibroblast)	ND	-	ND	ND	ND	[44]
MelJuso (melanoma)	ND	-	-	ND	ND	Unpublished
MO7e (acute myeloid leukemia)	-	ND	ND	ND	ND	[12]
Namalwa (Burkitt lymphoma)	-	-	-	+	++	Unpublished
NB1 (neuroblastoma)	-	++	++	++	++	[12]/This study
PC3 (prostate cancer)	ND	-	ND	ND	ND	Unpublished
Raji (Burkitt lymphoma)	-	++	++	++	++	This study
Ramos (Burkitt lymphoma)	-	+	++	ND	++	Unpublished
RPMI-8226 (myeloma)	-	-	ND	ND	ND	Unpublished
SAOS (osteosarcoma)	ND	-	ND	ND	ND	[46]
SkMel30 (melanoma)	ND	-	-	ND	ND	Unpublished
SK-N-Be(2)c (neuroblastoma)	-	ND	ND	ND	ND	[12]
SKW6.4 (transformed B-lymphoblastoid)	-	++	++	++	++	Unpublished
TC252 (Ewing sarcoma)	-	ND	ND	ND	ND	[12]
THP-1 (acute myeloid leukemia)	-	-	++	++	++	[12] / Unpublished
U2OS (osteosacroma)	-	-	-	-	-	[7] / Unpublished
Vero (monkey kidney)	-	-	-	ND	+	Unpublished
WM1366 (melanoma)	ND	+	++	ND	++	Unpublished
WM3248 (melanoma)	ND	+	++	ND	++	Unpublished

ND: not determined. -: not killed. +: < 20% killed. ++: > 20% killed.

**Figure 1:**
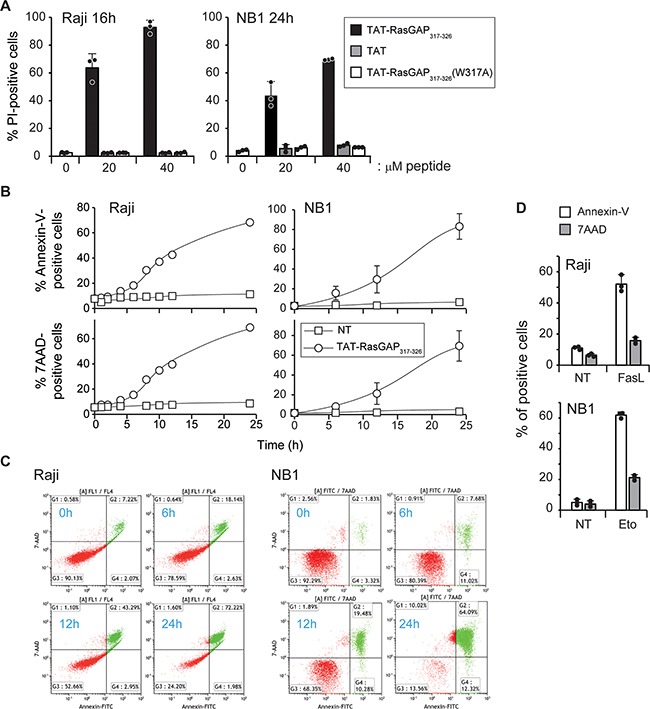
TAT-RasGAP_317-326_ directly kills Raji and NB1 cells **A.** Raji cells and NB1 cells were treated for 16 and 24 hours, respectively, with 0, 20 and 40 μM TAT-RasGAP_317-326_, TAT or TAT-RasGAP_317-326_(W317A). Cell death, corresponding to the percentage of propidium iodide (PI)-positive cells, was determined by flow cytometry. Results correspond to the mean +/− 95% confidence interval (CI) of 3 independent experiments. **B.** Raji and NB1 cells were treated with 20 μM and 40 μM TAT-RasGAP_317-326_, respectively, for the indicated periods of time. Phosphatidylserine exposure and plasma membrane permeabilization were then analyzed by flow cytometry using Annexin-V and 7AAD staining, respectively. **C.** Double stain analysis of 7AAD- and Annexin-V positive cells in Raji and NB1 cells after the indicated periods of time of TAT-RasGAP_317-326_ treatment. **D.** Raji and NB1 cells were treated with 150 ng/mL FasL for 16 hours and 10 μg/mL etoposide for 9 hours, respectively. Cell death was analyzed as in panel B. The results correspond to the mean +/− 95% CI of 3 independent experiments.

Two of the tumor cells that were efficiently killed by the peptide, the Raji Burkitt lymphoma and the NB1 neuroblastoma cell lines, were used to investigate the manner by which the peptide induced death. Supplementary Figure S2 shows that the peptide did not alter the proportion of cells in a given cell cycle stage, suggesting that cell cycle regulators are not targeted by the peptide. When treated with an inactive point mutant of the peptide [TAT-RasGAP_317-326_ (W317A)] [13], or with the TAT cell-penetrating peptide alone, viability of Raji and NB1 cells was not affected (Figure 1A). This demonstrates that the direct killing ability of TAT-RasGAP_317-326_ is not carried solely by the TAT moiety but depends on specific RasGAP sequences. TAT-RasGAP_317-326_ induced membrane permeabilization in a dose-dependent manner in both Raji and NB1 cells (Supplementary Figure S3A). The kinetic of death, induced by the peptide was assessed using Annexin-V and 7AAD staining. Annexin-V binds to phosphatidylserine (PS) exposed at the cell surface, a phenomenon that is generally characteristic of apoptosis. The 7AAD dye is plasma membrane impermeable and thus only labels cells with compromised cell permeability, which is typically seen in necrotic cells. Apoptotic cells bind Annexin-V but remain initially 7AAD negative [14]. Figure 1B-1C shows that Annexin-V and 7AAD positivity occurred concomitantly in Raji and NB1 cells when incubated with TAT-RasGAP_317-326_. In contrast and as expected, apoptotic inducers in these cells (e.g. Fas ligand (FasL) or etoposide) induced PS exposure before membrane impermeability was compromised (Figure 1D). Of note, in both Raji and NB1 cells, TAT-RasGAP_317-326_ induced a drop in mitochondrial membrane potential (Supplementary Figure S3B).

Reactive oxygen species (ROS) production upon peptide treatment was investigated. Intracellular hydrogen peroxide (H_2_O_2_) and cytosolic superoxide (CO_2_^.−^) were not augmented by TAT-RasGAP_317-326_ (Supplementary Figure S4A). The sensors for these ROS were functional as shown in Jurkat cells treated with APO866, a nicotinamide phosphoribosyltransferase inhibitor (Supplementary Figure S4B). In contrast to hydrogen peroxide and cytosolic superoxide, mitochondrial superoxide (mO_2_^.−^) was increased in NB1 and, less markedly, in Raji cells in response to TAT-RasGAP_317-326_ treatment (Supplementary Figure S4A). To assess the impact of mitochondrial ROS production on the viability of these cells, they were incubated with MitoTEMPO, a mitochondria-specific ROS scavenger. Supplementary Figure S4C shows that MitoTEMPO efficiently reduced TAT-RasGAP_317-326_-induced mO_2_^.−^ production in NB1 cells and this lowered death induced by the peptide. However, in Raji cells, MitoTEMPO did neither reduce the level of mO_2_^.−^ nor TAT-RasGAP_317-326_-induced death. While it is not clear why MitoTEMPO failed to inhibit mitochondrial superoxide production in Raji cells, the data obtained in NB1 cells indicate that TAT-RasGAP_317-326_ might kill some cells via production of mitochondrial ROS.

As the RasGAP-derived peptide impacted the functionality of mitochondria, we determined if it modulated cellular ATP levels. As shown in Supplementary Figure S4D, the peptide induced a drop in ATP levels in Raji and NB1 cells. Intriguingly, this drop started to occur before any detectable cell death, suggesting that the peptide impacts cellular metabolism before inducing membrane permeabilization.

**Figure 2:**
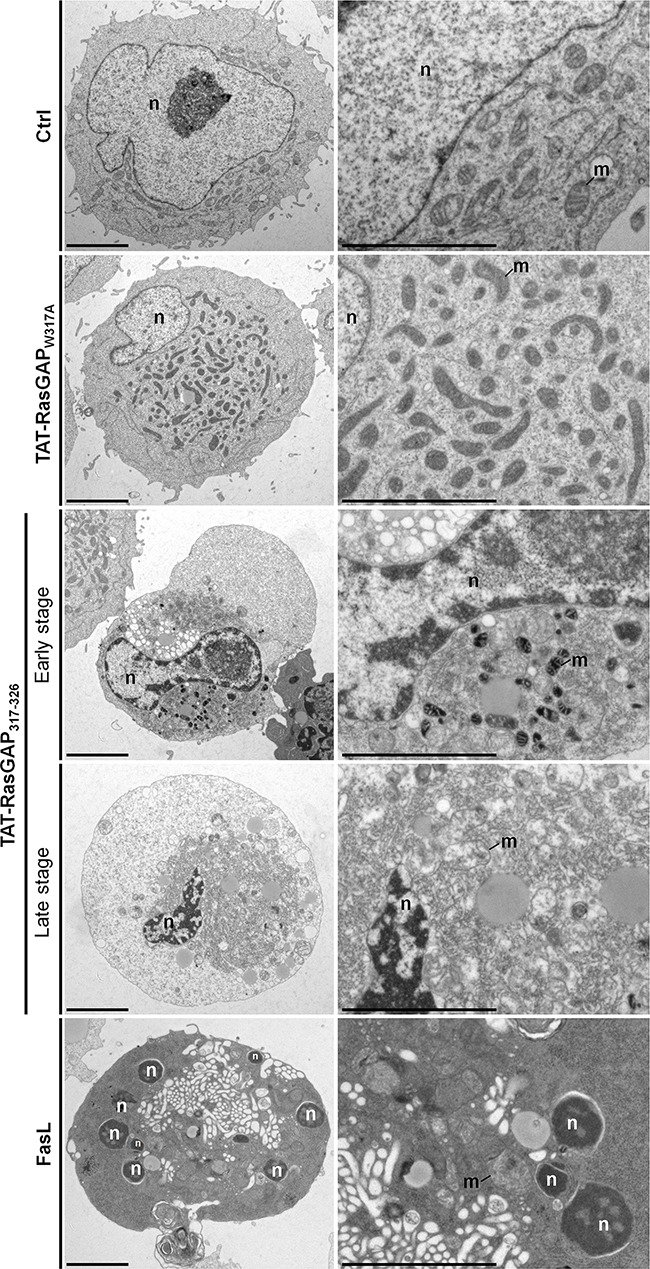
Ultrastructural analysis of TAT-RasGAP_317-326_-induced cell death in Raji cells Representative electron micrographs showing the morphological ultrastructural features in Raji cells left untreated (Ctrl) or incubated with 20 μM TAT-RasGAP_317-326_ (W317) (24 hours), 20 μM TAT-RasGAP_317-326_ (24 hours) or with 150 ng/mL FasL (16 hours). Scale bars: 5 μm. n: nucleus; m: mitochondria.

**Figure 3:**
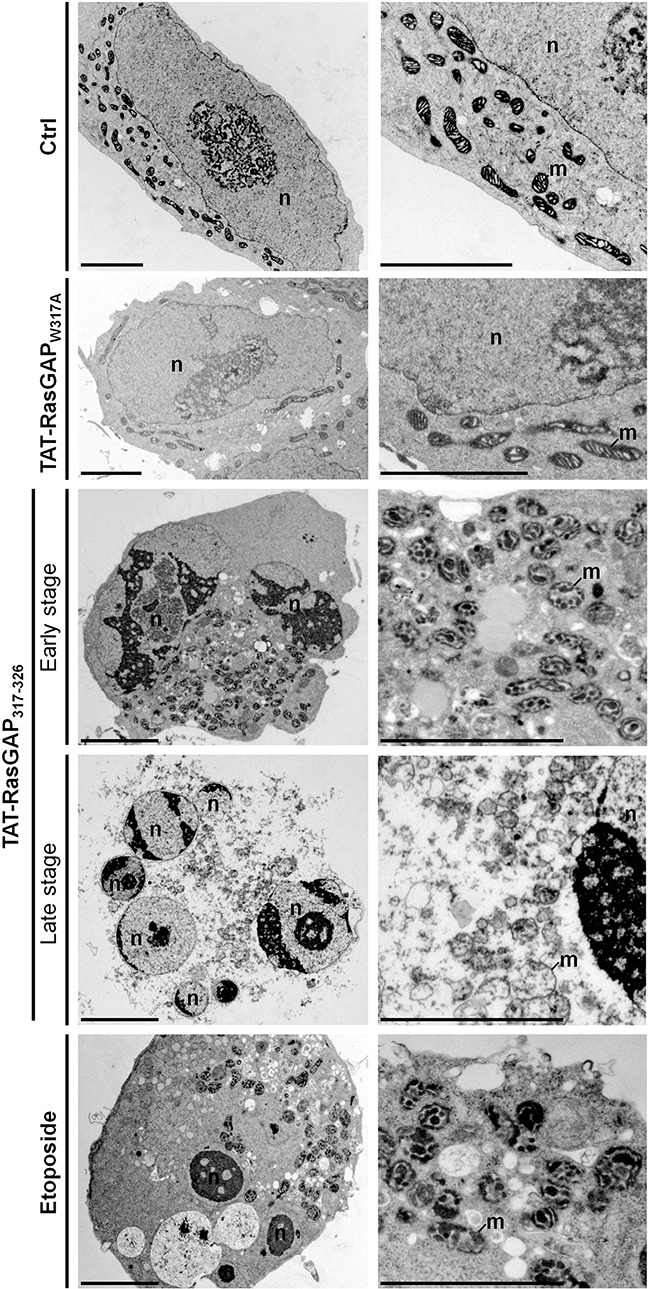
Ultrastructural analysis of TAT-RasGAP_317-326_-induced cell death in NB1 cells Representative electron micrographs showing the morphological ultrastructural features in NB1 cells in control condition (Ctrl) or after 40 μM TAT-RasGAP_317-326_ (w317A) (24 hours), 40 μM TAT-RasGAP_317-326_ (24 hours) or 10 μg/mL etoposide (8 hours) treatments. Scale bar: 5 μm. n: nucleus; m: mitochondria.

To evaluate the morphological changes involved in TAT-RasGAP_317-326_-induced cell death, we performed ultrastructural analyses in Raji and NB1 cells using electron microcopy (Figures 2 and 3). To have a comparison with apoptosis, we also treated Raji cells with FasL, a well-known apoptotic inducer (Figure 2). FasL treatment induced apoptotic cell death with classical morphological criteria including nuclear and cytoplasmic condensation, chromatin condensation throughout the nucleus (pyknosis), nuclear fragmentation, and minimal alterations of organelles (including mitochondria). TAT-RasGAP_317-326_-treated Raji cells displayed different morphological features than those seen in FasL-treated cells (Figure 2). At first, slight and heterogeneous chromatin condensation without nuclear fragmentation, shrinkage of the cytoplasm and accumulation of cytoplasmic materials around the nucleus were observed. Organelles aggregated in distinct portions of the cytoplasm whereas some cytoplasmic areas seemed to be devoid of organelles. Organelles displayed a relatively good preservation but some mitochondria showed condensed features. Then, at later stages, Raji cells displayed a necrotic-like phenotype such as huge organelle swelling and loss of plasma membrane integrity. The inactive TAT-RasGAP_317-326_ (W317A) mutant did not alter the ultrastructural morphology of Raji cells.

Whereas TAT-RasGAP_317-326_-induced Raji cell death displayed a non-canonical morphological phenotype, TAT-RasGAP_317-326_-induced NB1 cell death showed some morphological features of apoptosis including chromatin condensation, nuclear fragmentation and condensed mitochondria at early stage and then secondary necrosis morphological features at later stage (Figure 3). Morphological features in TAT-RasGAP_317-326_-induced NB1 cell death are therefore relatively similar to those observed when NB1 cells were exposed to the pro-apoptotic drug etoposide. However, clear differences were detected at the ultrastructural level between TAT-RasGAP_317-326_- and etoposide-treated NB1 cells. For example, the peptide never induced pyknosis in NB1 cells while etoposide almost always did so. Moreover, cytoplasmic materials often accumulated close to the nucleus in response to TAT-RasGAP_317-326_, while this was rarely the case when cells were incubated with etoposide. As seen in Raji cells, the inactive TAT-RasGAP_317-326_ (W317A) mutant had no effect on the ultrastructure of NB1 cells.

The data shown in Figures 2 and 3 indicate that TAT-RasGAP_317-326_ is inducing a form of death that is sharing some characteristics with apoptosis, at least in some cell lines, but which does not seem to be strictly equivalent to this mode of death. We therefore aimed to determine if other types of cell death were triggered by the RasGAP-derived peptide.

### Apoptosis inhibition does not protect against TAT-RasGAP_317-326_-induced cell death in Raji cells and only partially in NB1 cells

**Figure 4:**
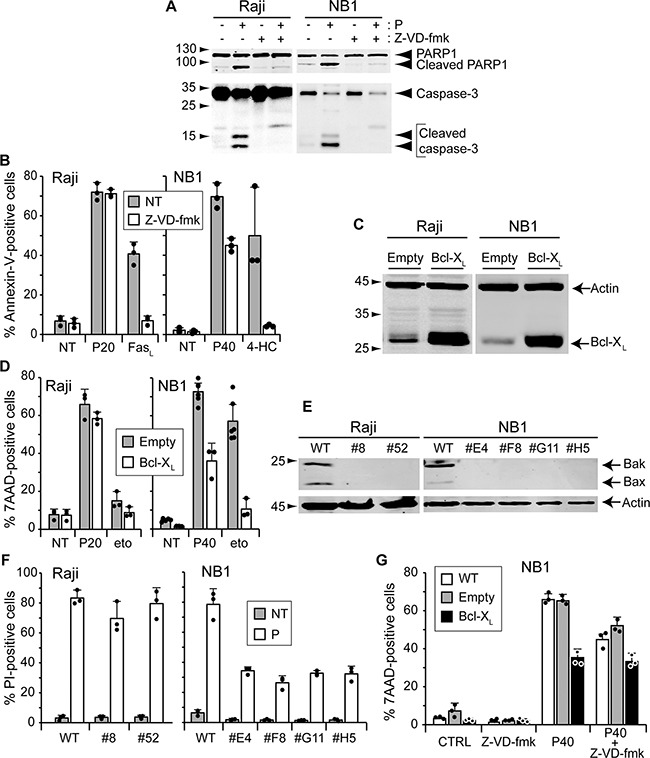
Inhibition of apoptosis does not prevent TAT-RasGAP_317-326_-induced cell death **A.** Raji cells and NB1 cells were pre-incubated or not 1 hour with 10 μM of the pan-caspase inhibitor Z-VD-fmk. Raji cells were then treated with 20 μM TAT-RasGAP_317-326_ for 16 hours and NB1 cells with 40 μM TAT-RasGAP_317-326_ for 24 hours. Cells were lysed and cleavage of PARP and caspase-3 analyzed by Western blotting. **B.** Raji and NB1 cells were pre-incubated or not 1 hour with 10 μM of the pan-caspase inhibitor Z-VD-fmk. Raji cells were then treated with 20 μM TAT-RasGAP_317-326_ (P20) and 150 ng/mL Fas-ligand (FasL) for 16 hours. NB1 cells were treated with 40 μM TAT-RasGAP_317-326_ (P40) and 30 μM 4-HC, the active form of cyclophosphamide, for 24 hours. Cell death (corresponding to the % of Annexin-V positive cells) was determined by FACS. Results correspond to the mean +/− 95% CI of 3 independent experiments. **C.** Raji and NB1 cells were infected with empty viruses or viruses encoding Bcl-X_L_. Bcl-X_L_ expression levels were assessed by Western blotting. **D.** Raji cells overexpressing or not Bcl-X_L_ were treated with 20 μM TAT-RasGAP_317-326_ and 250 μM etoposide (eto) for 16 hours. NB1 cells overexpressing or not Bcl-X_L_ were treated with 40 μM TAT-RasGAP_317-326_ and 50 μM etoposide for 24 hours. Cell death (corresponding to the % of 7AAD positive cells) was determined by FACS. The results correspond to the mean +/− 95% CI of at least three independent experiments. **E.** Bax and Bak were disrupted in Raji and NB1 cells using the CRISPR/Cas9 technology. Loss of expression was confirmed by Western blotting. **F.** Wild-type and Bax/Bak double-knock-out Raji and NB1 cells were treated with 20 μM TAT-RasGAP_317-326_ for 16 hours and 40 μM TAT-RasGAP_317-326_ for 24 hours, respectively. Cell death (corresponding to the % of PI-positive cells) was determined by FACS. Results correspond to the mean +/− 95% CI of 3 independent experiments. **G.** NB1 cells overexpressing or not Bcl-X_L_ were pre-incubated or not 1 hour with 10 μM of the pan-caspase inhibitor Z-VD-fmk and then treated with 40 μM TAT-RasGAP_317-326_. After 24 hours incubation, cell death (corresponding to the % of 7AAD-positive cells) was determined by FACS.

TAT-RasGAP_317-326_ induced caspase-3 activation and PARP1 cleavage in both Raji and NB1 cells (Figure 4A), indicating that the peptide can trigger an apoptotic program. However, inhibition of caspase activity with the pan-caspase Z-VD-fmk inhibitor [15, 16] (Figure 4A), while efficiently blocking apoptosis triggered by FasL, had no effect on death induced by TAT-RasGAP_317-326_ in Raji cells (Figure 4B). In NB1 cells, however, a partial protection was observed (Figure 4B). Intrinsic mitochondrial apoptosis is characterized by the release of cytochrome *c* into the cytosol, which depends on the pore forming proteins Bax and Bak. Unlike tBid, TAT-RasGAP_317-326_ did not induce cytochrome *c* release from isolated mitochondria (Supplementary Figure S5), suggesting no direct action at the mitochondria level. The activity of Bax and Bak can be inhibited by over-expression of anti-apoptotic Bcl-2 family members, such as Bcl-X_L_ [17, 18]. Bcl-X_L_ over expression (Figure 4C) partially prevented TAT-RasGAP_317-326_-induced apoptosis in NB1 cells (Figure 4D). In Raji cells, etoposide, even at high doses, only induced a slight increase in apoptosis, most probably because Raji cells express high basal level of Bcl-2 [19]. However, Bcl-X_L_ overexpression did not protect Raji cells against TAT-RasGAP_317-326_-induced death (Figure 4D). Similar results were obtained when Bax and Bak expression were removed by gene disruption using the CRISPR/Cas9 technology (Figure 4E-4F). Combining Bcl-X_L_ overexpression and Z-VD-fmk treatment did not induce a stronger inhibition of TAT-RasGAP_317-326_-induced death in NB1 cells as compared to individual inhibitor applications (Figure 4G).

### TAT-RasGAP_317-326_ does not trigger necroptosis

**Figure 5:**
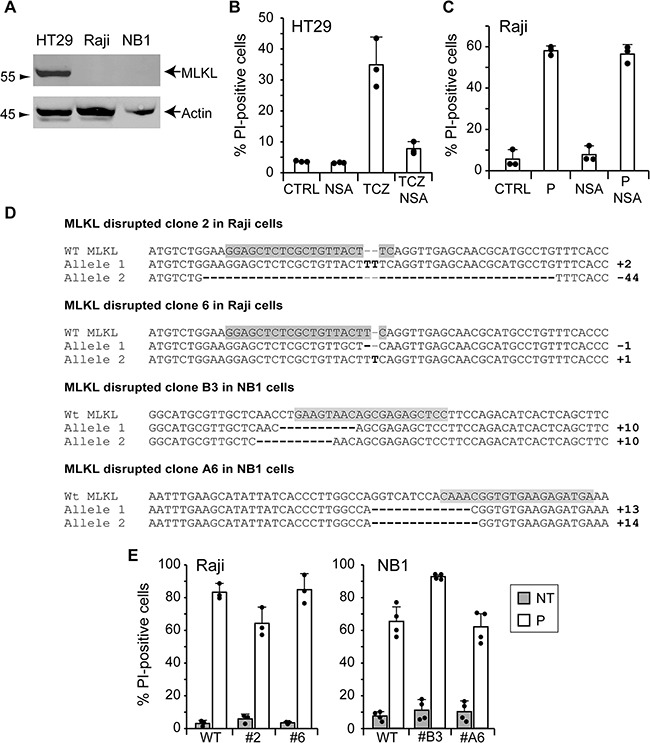
TAT-RasGAP_317-326_-induced cell death does not require the necroptosis machinery **A.** Expression of MLKL in wild-type HT29 cells, Raji cells and NB1 cells assessed by Western blotting. **B.** HT29 cells were pretreated for 1 hour with 10 μM necrosulfonamide (NSA) and necroptosis was induced by the addition of 30 ng/ml TNF-α (T), 2 μg/ml cycloheximide (C) and 10 μM Z-VD-fmk (Z). After 24 hours, cell death was determined by FACS after staining with PI. **C.** Raji cells were pretreated for 1 hour with 10 μM NSA before the addition of 20 μM TAT-RasGAP_317-326_ (P). After 16 hours incubation, cell death (corresponding to the % of PI-positive cells) was determined by FACS. The results correspond to the mean +/− 95% CI of three independent experiments. **D.** DNA sequences of wild-type MLKL gene and MLKL alleles of clones 2 and 8 and clones B3 and A6 of Raji cells and NB1 cells, respectively. Differences with the wild-type MLKL sequence are highlighted in bold. The sgRNAs directed against MLKL are highlighted in grey. **E.** Wild-type and MLKL disrupted Raji and NB1 cells were treated for 16 and 24 hours, respectively. Cell death (corresponding to the % of PI-positive cells) was measured by FACS. The results correspond to the mean +/− 95% CI of minimum three independent experiments.

As apoptosis was not, or only partially, involved in the death induced by TAT-RasGAP_317-326_, we investigated whether other forms of death could be involved. Necroptosis, also called programmed necrosis, is a form of cell death that differs from apoptosis at morphological and signaling levels [20, 21]. It is characterized by cell rounding, gain in cell volume, organelle swelling and plasma membrane rupture. Necroptosis requires receptor-interacting protein (RIP) 1 and 3. The downstream target of the complex formed by RIP1/RIP3 was identified as mixed lineage kinase domain-like protein (MLKL) [22, 23]. Activation of MLKL leads to its translocation from the cytosol to plasma and intracellular membranes, and subsequent loss of membrane integrity [24]. In cells such as the HT29 colorectal adenocarcinoma, necroptosis can be triggered by tumor necrosis factor alpha (TNF-α) stimulation when caspases and translation are inhibited [25]. We were however unable to induce Raji and NB1 necroptosis using this protocol. This could be the consequence of a low MLKL expression (Figure 5A). To assess the involvement of necroptosis in TAT-RasGAP_317-326_-induced death, Raji cells were treated with necrosulfonamide (NSA), an MLKL inhibitor [23]. NSA efficiently prevented necroptosis in HT29 cells (Figure 5B) but had no effect on the death provoked by the RasGAP-derived peptide (Figure 5C). One could argue that NSA might not be efficient in Raji cells, even at concentrations shown to be efficient in sensitive cell lines such as HT29. We therefore knocked out MLKL in Raji and NB1 cells as another approach to prevent necroptosis. Because endogenous levels of MLKL in these cells were low and could not be detected in Raji and NB1 cells (Figure 5A), the targeted DNA region by the Cas9 endonuclease was sequenced. Figure 5D shows that both alleles of Raji clones #2 and #6 and NB1 clones #B3 and #A6 were disrupted, engendering frameshift mutations. These MLKL disrupted clones were then treated with the TAT-RasGAP_317-326_ peptide but this did not prevent cell death (Figure 5E). Collectively, these data demonstrate that TAT-RasGAP_317-326_ does not require the molecular machinery of necroptosis to kill Raji and NB1 cells.

### TAT-RasGAP_317-326_ does not trigger pyroptosis

**Figure 6:**
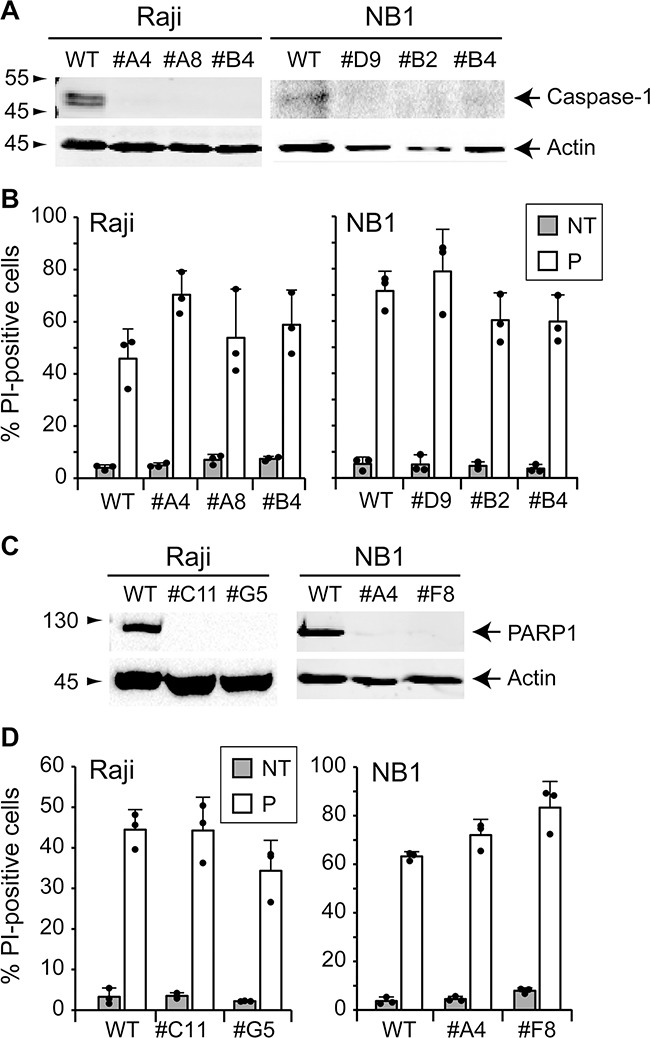
TAT-RasGAP_317-326_-induced cell death is caspase-1- and PARP1-independent **A.** Caspase-1 was disrupted in Raji and NB1 cells using the CRISPR/Cas9 technology. Loss of expression was confirmed by Western blotting. **B.** Wild-type and caspase-1 knock-out Raji cells were treated or not with 20 μM TAT-RasGAP_317-326_ (P) for 16 hours. Wild-type and caspase-1 knock-out NB1 cells were treated or not with 40 μM TAT-RasGAP_317-326_ (P) for 24 hours. Cell death was then assessed by flow cytometry after PI staining. Results correspond to the mean +/− 95% CI of 3 independent experiments. **C.** PARP1 was disrupted in wild-type Raji and NB1 cells using CRISPR/Cas9 technology. Loss of expression was confirmed by Western blotting. **D.** Wild-type and PARP1 knock-out Raji cells were treated or not with 20 μM TAT-RasGAP_317-326_ (P) for 16 hours. NB1 cells were treated or not with 40 μM TAT-RasGAP_317-326_ (P) for 24 hours. Cell death was then assessed by flow cytometry after PI staining. Results correspond to the mean +/− 95% CI of 3 independent experiments.

We next examined the implication of pyroptosis. This form of programmed cell death is stimulated by microbial and viral infections but also by stroke and cancer [26]. Morphological features displayed by pyroptotic cells are common with apoptosis and/or necrosis [26, 27]. Defined as a caspase-1-dependent cell death, pyroptosis results in the production of inflammatory cytokines such as interleukin-1β (IL-1β) and IL-18 and ends up in cell lysis. To determine whether pyroptosis is a type of cell death induced by TAT-RasGAP_317-326_, Raji and NB1 cells lacking caspase-1 were generated. Loss of caspase-1 expression was confirmed in different clones by Western blotting (Figure 6A). The ability of the peptide to cause cell death was not abrogated in caspase-1 knock-out Raji and NB1 cells (Figure 6B). This is in line with the caspase inhibition results shown above (Figure 4B) as Z-VD-fmk is also expected to prevent caspase-1 activity [16]. Altogether, this indicates that the RasGAP-derived peptide does not elicit pyroptosis.

### TAT-RasGAP_317-326_ does not trigger parthanatos

Parthanatos is a cell death mode that is initiated by over-activation of poly (ADP-ribose)-polymerase 1 (PARP1). Under physiological conditions, PARP1 is involved in DNA repair. To maintain genomic homeostasis, PARP1 detects single strand DNA breaks, uses NAD^+^ to synthetize poly (ADP-ribose) (PAR) and attaches PAR on itself and other target proteins [28, 29]. This leads to the recruitment of critical proteins for DNA repair [30, 31]. Hyperactivation of PARP1 contributes to NAD^+^ and ATP depletion and translocation of apoptosis-inducing factor (AIF) from the mitochondria to the nucleus [32–34]. To investigate if TAT-RasGAP_317-326_ triggers parthanatos, PARP1 knock-out cells were generated. Loss of PARP1 expression was controlled by Western blotting (Figure 6C). Figure 6D shows that in the absence of PARP1, Raji and NB1 cells are still killed by the peptide. Hence, the peptide does not trigger parthanatos.

### Autophagy and TAT-RasGAP_317-326_-induced death

**Figure 7:**
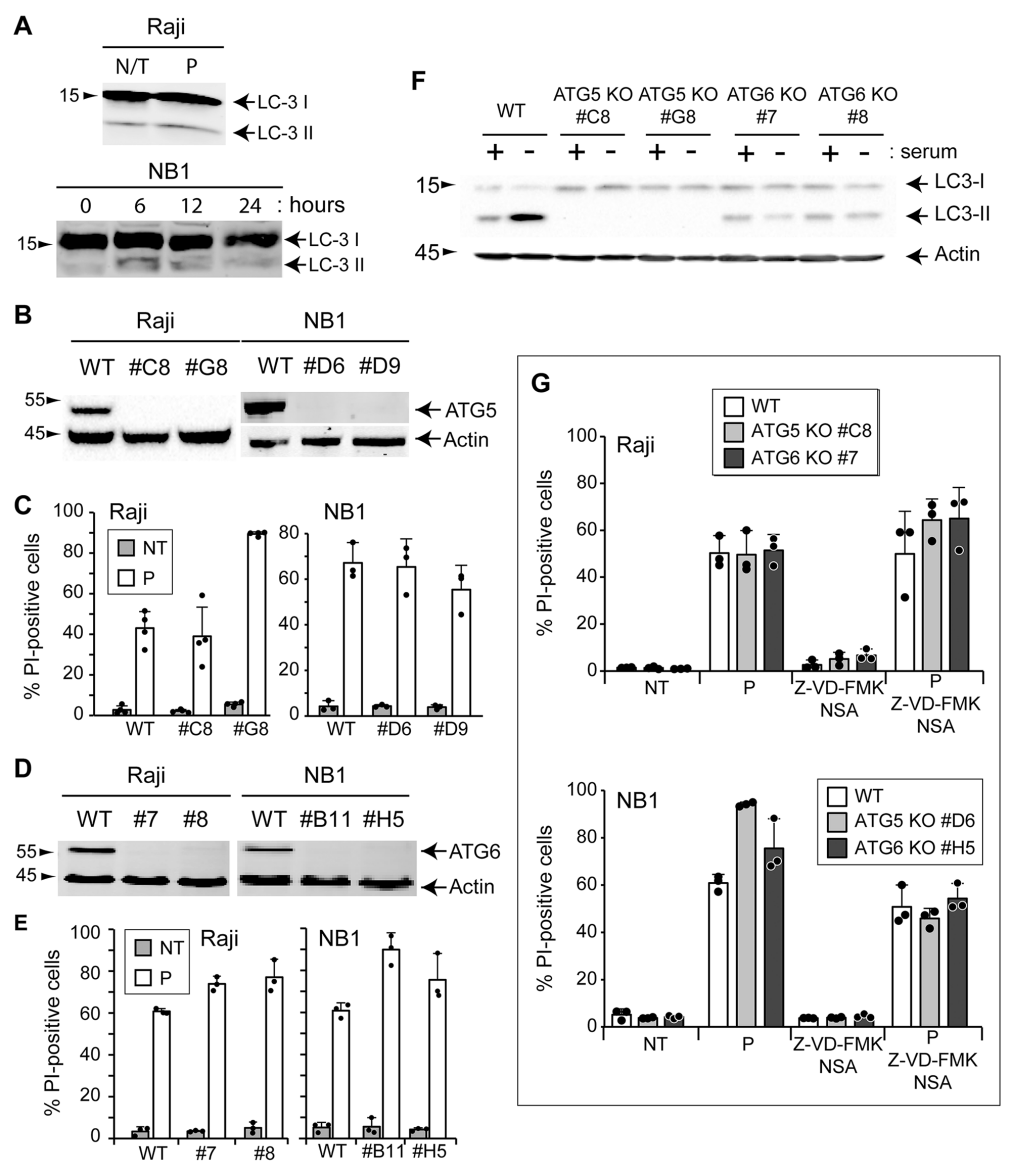
Autophagy is not involved in TAT-RasGAP_317-326_-induced cell death **A.** Raji cells were treated during 16 hours with 20 μM TAT-RasGAP_317-326_ (P) or not (N/T) and LC3 expression was analyzed by Western blotting. NB1 cells were treated during the indicated periods of time with 40 μM TAT-RasGAP_317-326_ and LC3 expression was analyzed by Western blotting. **B.** and **D.** ATG5 and ATG6 were individually disrupted in Raji and NB1 cells using the CRISPR/Cas9 technology. Loss of expression was confirmed by Western blotting. **C.** Wild-type and ATG5 knock-out Raji cells were treated or not with 20 μM TAT-RasGAP_317-326_ (P) for 16 hours. Wild-type and ATG5 knock-out NB1 cells were treated or not with 40 μM TAT-RasGAP_317-326_ (P) for 24 hours. Cell death was then assessed by flow cytometry after PI staining. Results correspond to the mean +/− 95% CI of 3 independent experiments. **E.** Wild-type and ATG6 knock-out Raji and NB1 cells were treated as described in panel D. Cell death was then assessed by flow cytometry after PI staining. Results correspond to the mean +/− 95% CI of 3 independent experiments. **F.** Wild-type, ATG5 knock-out and ATG6 knock-out Raji cells were cultured for 48 hours in presence or absence of serum. LC3 conversion was analyzed by Western blotting. **G.** Wild-type, ATG5 knock-out and ATG6 knock-out Raji cells were pretreated or not with 10 μM Z-VD-fmk and 10 μM necrosulfonamide (NSA) for 1 hour and then treated or not with 20 μM TAT-RasGAP_317-326_ (P) for 16 hours. Wild-type, ATG5 knock-out and ATG6 knock-out NB1 cells were pretreated or not with 10 μM Z-VD-fmk and 10 μM necrosulfonamide (NSA) for 1 hour and then treated or not with 40 μM TAT-RasGAP_317-326_ (P) for 24 hours. Cell death was then assessed by flow cytometry after PI staining. Results correspond to the mean +/− 95% CI of 3 independent experiments.

Autophagy is a process of self-degradation. During starvation, it allows cells to maintain energy levels via the degradation and recycling of cellular cytoplasmic constituents, allowing cell survival. Autophagosome formation involves the lipidation of the LC3 protein [35]. This pro-survival function of autophagy is well accepted [36, 37]. Autophagy may in some conditions trigger cell death [38]. To test if the peptide modulates autophagy, the autophagic marker lipidatino of LC3 was examined by Western blotting. Figure 7A shows that conversion of the LC3-I unlipidated form to the LC3-II lipidated form is similar in untreated cells and in cells treated with TAT-RasGAP_317-326_. To rule out the involvement of autophagy in TAT-RasGAP_317-326_-triggered death, two autophagic genes, ATG5 and ATG6, were disrupted (Figures 7B and 7D). Disruption of ATG5 fully prevented autophagy in Raji cells, as assessed by the absence of LC3 lipidation in serum deprivated conditions (Figure 7F). Moreover, autophagy induced by serum starvation fully prevented in cells lacking ATG6 (Figure 7F). However, the absence of ATG5 and ATG6 did not prevent TAT-RasGAP_317-326_-induced cell death in Raji and NB1 cells (Figures 7C and 7E), suggesting that autophagy plays no role in TAT-RasGAP_317-326_-mediated death.

It is possible that the peptide triggers cell death via the activation of several pathways in parallel. We therefore investigated the effect of multiple cell death inhibition on TAT-RasGAP_317-326_ toxicity. Figure 7G shows that inhibition of apoptosis and necroptosis in autophagy-deficient Raji and NB1 cells did not protect against the cytotoxic effect of the peptide. Taken together, these results indicate that none of the “classical” death pathways mediate the killing activity of TAT-RasGAP_317-326_.

## DISCUSSION

Apoptosis had been the most intensively studied mode of regulated cell death for years. Thus, apoptotic inducers were and are largely used in the clinic as cancer therapies. However, tumor cell resistance to apoptosis, leading to cancer progression, is currently a major clinical problem. Some strategies could overcome this problem. For example, restoring the sensitivity of cancer cells to apoptosis might improve the efficacy of anti-tumor drugs. Another possibility would be to trigger alternate modes of death to which cancer cells are not resistant. The results presented here indicate that the TAT-RasGAP_317-326_ peptide has the capacity to kill some tumor cells in a manner distinct from the known characterized forms of death.

Our data show that necroptosis, autophagy, parthanatos and pyroptosis are neither activated nor involved in the toxicity induced by TAT-RasGAP_317-326_. In contrast, the apoptotic pathway is efficiently stimulated by the peptide. However, inhibiting apoptosis, either pharmacologically or genetically, did not (Raji cells), or only partially (NB1 cells), prevent TAT-RasGAP_317-326_ cytotoxicity. Hence, TAT-RasGAP_317-326_ has the potential to activate apoptosis and another form of cell death that is distinct from the above mentioned death pathways. The ability of triggering multiple forms of death has been reported for other compounds. Indeed, shikonin and cisplatin, depending on the concentrations used, stimulate either apoptosis or necroptosis/necrosis [39, 40]. Another example is the lipophilic mitochondria-targeted F16 compound that was shown to elicit death via apoptosis [41]. However, Bcl-2 overexpression did not block the capacity of F16 to trigger a necrotic cell death, demonstrating that one compound can have a dual ability to kill through both apoptosis and necrosis.

Our results clearly indicate that TAT-RasGAP_317-326_ has the potential to stimulate both apoptosis and an alternative form of death in tumor cells of various origins. However, it is currently not known whether these two forms of death are activated independently or whether the triggering event leading to the alternative form of death has the capacity to also stimulate apoptosis.

In Raji cells, the sensitivity to the alternative form of death stimulated by TAT-RasGAP_317-326_ is seemingly high and the apoptotic pathway can be blocked without affecting the overall death response. In NB1 cells, activation of the alternate form of death appears suboptimal and efficient killing requires concomitant activation of apoptosis. Consequently, it seems that there is a continuum of sensitivities among cancer cell lines to the direct killing action of TAT-RasGAP_317-326_.

Only a fraction of the tested cell lines were found to be sensitive to the killing activity of the peptide. To assess if those cells share common biological features that were not present in the resistant cell lines, we performed mutational and transcriptomics bioinformatical analyses on six sensitive and six resistant cell lines (subjected to 40 μM TAT-RasGAP_317-326_). Supplementary Figure S6A lists the 6 genes with the strongest differential mutational status between resistant and sensitive cell lines. Even in these genes, there was no strict association between the mutation status and the sensitivity of the cell lines to the peptide. It is unlikely that these genes, individually at least, drive the peptide sensitivity of a cell line. In agreement with this is the absence of correlation between their expression and the sensitivity of a cell line to be killed by the peptide (Supplementary Figure S6B). Moreover, there is no statistical support for a difference in the overall mutational rate of these cell lines (Supplementary Figures S6C-S6D). Gene profiling analysis revealed that cell lines cluster according to their origin rather than their sensitivity to TAT-RasGAP_317-326_ (Supplementary Figure S6E). We finally performed a differential expression analysis to find whether the expression of certain genes was specifically associated with TAT-RasGAP_317-326_ sensitivity but failed to detect any (Supplementary Figure S6F). These results suggest that neither mutation nor transcriptional regulation are involved in the regulation of TAT-RasGAP_317-326_ sensitivity. However, we should take into account that the cell lines were not always tested in identical experimental conditions (e.g. different culture media used for the experimentation). Moreover, as the peptide requires entry into cells via the HIV-TAT_48-57_ portion, it is also possible that variations in peptide intake could explain some of the differences in the sensitivity observed between the cell lines.

At present, we cannot rule out the possibility that the alternate form of death triggered by the peptide is a form of necrosis. One hypothesis that we cannot dismiss is that TAT-RasGAP_317-326_ enters cells and then once in the cytoplasm alters, directly or indirectly, plasma membrane integrity by interacting with specific molecules that are enriched in the inner leaflet of the membrane. This mode of action has been reported for defensin NaD1, a host defense peptide [42]. NaD1 was shown to enter mammalian cells and bind to phosphatidylinositol 4,5-bisphosphate (PIP2), leading to rapid membrane destabilization and permeabilization. As highlighted in this example, cell death induced by a peptide may not, or not only, depend on protein binding but also on specific membrane lipid interaction.

To conclude, our finding could potentially have interesting clinical relevance since current anti-cancer therapies are mostly based on drugs that induce tumor cell apoptosis. Consequently, determining that a peptide is able to induce a distinct form of death in tumor cells could lead to the generation of innovative anti-cancer drugs that complement or be combined with existing ones.

## MATERIALS AND METHODS

### Cell lines

All cell lines were cultured in 5% CO_2_ at 37°C. 293T, 501Mel, PC3, HT29, U2OS and Vero were cultured in DMEM (Invitrogen, ref. no. 61965) supplemented with 10% heat-inactivated fetal bovine serum (FBS; Invitrogen, ref. no. 10270-106). Raji, A375, Daudi, HeLa, IGr37, IPC298, Jurkat, MelJuso, Namalwa, Ramos, RPMI-8226, SKMel30, SKW6.4, THP-1, WM1366 and WM3248 were cultured in RPMI (Invitrogen, ref. no. 61870) supplemented with 10% FBS. The NB1 neuroblastoma cells were maintained in neural basic medium composed of DMEM/F12 (Invitrogen, ref. no. 31331-028) supplemented with 2% B27 serum-free supplement (Invitrogen, ref. no. 17504044), 20 ng/ml human recombinant basic fibroblast growth factor (bFGF) (Peprotech, ref. no. 100-18B) and 20 ng/ml human recombinant epidermal growth factor (EGF) (Peprotech, ref. no. AF-100-15). Human peripheral blood lymphocytes (PBLs) were isolated by density centrifugation over a Ficoll-Paque gradient (Lymphoprep; Stemcell Technologies) from buffy coats of healthy human donors, obtained from the state of Vaud blood transfusion service. The donors gave written consent for potential use of their blood for medical research. B cells present in PBLs were positively stained with mouse FITC-labelled anti-CD19 antibody for 30 min at 4°C before flow cytometry analysis.

### Chemicals

TNFα and the protease inhibitor tablets were from Roche (ref. no. 11088939001 and 4693132001, respectively). Cycloheximide and etoposide were from Sigma (ref. no. C7698 and E1383 respectively). Necrosulfonamide was from Tocris bioscience (ref. no. 5025). The pan-caspase inhibitor Z-VD-fmk was a kind gift from Maxim Pharmaceuticals. Hexameric Fas ligand, resulting from the aggregation of 6 fusion proteins between Fas ligand and the Fc portion of IgG1 [43], was provided by Pascal Schneider (University of Lausanne). Puromycin was from Life technologies (ref. no. A11138-02) and 4-hydroperoxycyclophosphamide (4-HC) was from Niomech, (ref. no. D-18864).

### Peptides

TAT and TAT-RasGAP_317–326_ are retro-inverso peptides (i.e. synthesized with D-amino acids in the opposite direction compared to the natural sequence). The TAT moiety corresponds to amino acids 48–57 of the HIV TAT protein (RRRQRRKKRG) and the RasGAP_317–326_ moiety corresponds to amino acids 317–326 of the human RasGAP protein (DTRLNTVWMW). These two moieties are separated by two glycine linker residues in the TAT-Ras-GAP_317–326_ peptide. TAT-RasGAP_317-326_(W317A) has the tryptophan at position 317 mutated into an alanine. The peptides were synthesized at the department of biochemistry, University of Lausanne, Switzerland, using FMOC technology, purified by HPLC and tested by mass spectrometry.

### Cell death measurement

Cell death was measured with an Annexin-V-FITC / 7AAD kit (Beckman Coulter, ref. no. IM3614) or with propidium iodide (PI) (Sigma, ref. no. 81845) and used according to the manufacturer's instructions. Cells were scanned using a Beckman Coulter FC500 flow cytometer and data were analyzed with the Kaluza Version 1.3 software (Beckman Coulter).

### Cell cycle analysis

Cells were collected, washed once in PBS and fixed in 70 % ethanol at 4°C for 2 hours and then washed twice with PBS. DNA was stained with PI solution (10 μg/mL PI, 150 μg/mL RNase A in water) at 37°C for 30 minutes. Samples were analyzed by flow cytometry using a Beckman Coulter FC500 flow cytometer.

### TMRM staining

Cells were collected, washed once in PBS and stained with 100 μL of 150 nM tetramethylrhodamine methyl ester (TMRM) solution by incubating during 20 min at 37°C. 500 μL of PBS were added and then transferred to a sample tube which was analyzed by flow cytometry using a Beckman Coulter FC500 flow cytometer.

### Mitochondria isolation and cytochrome *c* release

Cells were harvested in PBS and centrifuged 10 min at 1,000xg. Cells were then resuspended in isotonic mitochondrial buffer (MB) (10 mM HEPES pH 7.4, 210 mM mannitol, 70 mM sucrose, 1 mM EDTA supplemented with one tablet of protease inhibitor cocktail per 50 mL), broken by five passages through a 25G1 0.5- by 2.5-mm needle fitted on a 2 mL syringe and centrifuged at 1,500xg for 5 min. This procedure was repeated twice and supernatants from each step were pooled and centrifuged 5 min at 1,500xg. Supernatant was collected, centrifuged 5 min at 2,000xg and further centrifuged 10 min at 9,000xg. Pellet was resuspended in MB (100 μL), centrifuged 10 min at 7,000xg and the pellet, representing the mitochondrial fraction, was finally resuspended in a volume of 100 μL of MB. 40 μg of mitochondria were incubated in KCl buffer (10 mM HEPES pH 7.4; 125 mM KCl; 0.5 mM EGTA; 4 mM MgCl_2_; 5 mM KH_2_PO_4_) and left untreated or treated with 20 μM TAT-RasGAP_317–326_, 20 μM TAT-RasGAP_317–326_ (W317A) or 40 nM tBid for 30 min at 37°C. Samples were then centrifuged 5 min at 16,000xg and supernatant and pellet analyzed by Western blotting for the presence of cytochrome *c*.

### Antibodies

The rabbit anti-Bcl-X_L_, the rabbit anti-caspase-3, the rabbit anti-LC3, the mouse anti-PARP1, the rabbit anti-actin antibodies were from Cell Signaling (ref. no. 2764, 9662, 2775, 9546, 4970 respectively). The rabbit anti-ATG6, the rabbit anti-total Bax and the rabbit anti-total Bak were from Santa Cruz Biotechnology (ref. no. sc-11427, sc-493 and sc-832 respectively). The mouse anti-caspase-1 was from Adipogen (ref. no. AG-20B-0048). The rabbit anti-ATG5 was from Abcam (ref. no. ab108327). The rat anti-MLKL was from Merck Millipore (ref. no. MABC604). The mouse FITC-labelled anti-CD19 was from Beckman Coulter (ref. no. A07768).

### Detection of cellular and mitochondrial ROS

Intracellular levels of cytosolic and mitochondrial superoxide as well as H_2_O_2_ production were determined by flow cytometry using live-cell permeant-specific fluorogenic probes, dihydroethidium (DHE; Marker Gene Technologies Inc, ref. no MGT-M1241-M010), MitoSOX (Molecular Probes, ref. no M36008) and 6-carboxy-20,70-dichlorodihydrofluorescein diacetate (carboxy-H2DCFDA; Molecular Probes, ref. no C-400), respectively. DHE is oxidized to red fluorescent ethidium by cytosolic superoxide, MitoSOX is selectively targeted to mitochondria, where it is oxidized by superoxide and exhibits red fluorescence and carboxy-H2DCFDA becomes green-fluorescent when oxidized with intracellular H_2_O_2_. After drug treatment, cells were harvested and transferred to flow cytometry tubes and incubated with 5 μM of specific probe at 37°C for 30 min. Cells were washed twice with PBS, resuspended in 500 μL PBS and, for visualization of the intracellular fluorescence, probes were excited at 488 nm and fluorescence emission were analyzed by flow cytometry.

### Determination of intracellular ATP content

Intracellular ATP content was measured with the ATP determination kit (Molecular Probes, ref. no A22066) according to manufacturer's instructions. Briefly, after drug treatment, cells were collected, washed twice in PBS, resuspended in 100 μL lysis buffer (NaHCO_3_ 20 mM + Na_2_CO_3_ 100 mM) and kept at −80°C for at least 4h. Cell lysates and ATP standards (10 μL) were mixed with standard reaction solution (90 μL) and luminescence was determined using an automatized bioluminometer (Promega Glomax 96 microplate luminometer). ATP level for each sample was normalized to protein content.

### Plasmids

The lentiviral vector lentiCRISPRv2 [44] was obtained from Addgene (#868, Addgene, ref. no. 52961). The pMD2.G plasmid (#554, Addgene, ref. no. 12259) encodes the envelope of lentivirus. The psPAX2 plasmid (#842, Addgene, ref. no. 12260) encodes the packaging system. The hBclXL.LEGO-iG2 plasmid (#863) was constructed by subcloning the 771 bp EcoRI fragment from hBcl-XL. dn3 (#274) into LeGO-iG2 (#807; Addgene: plasmid 27341).

### Lentivirus production

Recombinant lentiviruses hBclXL.LEGO-iG2 were produced as described [45] with the following modifications: pMD.G (#218) and pCMVDR8.91 (#219)were replaced by pMD2.G and psPAX2 respectively.

### Genome editing by CRISPR method

Single guide RNAs targeting the early exon of the protein of interest were chosen in the sgRNA library [46] and are listed in Table 2. LentiCRISPR plasmids specific for a gene were created according to the provided instructions. Oligos were designed as follow: Forward 5′-CACCGnnnnnnnnnnnnnnnnnnnn-3′; Reverse-3′-CnnnnnnnnnnnnnnnnnnnnCAA-5′, where nnnnnnnnnnnnnnnnnnnn in the forward oligo corresponds to the 20 bp sgRNA. Oligos were synthetized, then phosphorylated and annealed to form oligo complexes. LentiCRISPR vector was BsmBI digested and dephosphorylated. Linearized vector was purified and gel extracted and ligated to oligo complexes. The lentiCRISPR vector containing the sgRNA was then used for lentivirus production. Cells were infected and selected with the appropriate dose of puromycin (2 μg/ml for Raji, 1 μg/ml for NB1). Clone isolation was performed by limiting dilution in 96 well-plate.

**Table 2:** List of sgRNAs used to disrupt the indicated target genes (and in which exons)

Target gene	sgRNA name	sgRNA sequence	Exon number
**ATG5**	sgATG5.3	AAGATGTGCTTCGAGATGTG	3
**ATG5**	sgATG5.5	AAGAGTAAGTTATTTGACGT	4
**ATG6**	sgATG6.1	ATTTATTGAAACTCCTCGCC	7
**ATG6**	sgATG6.2	ATCTGCGAGAGACACCATCC	7
**Bak**	sgBak.1	GCTCACCTGCTAGGTTGCAG	3
**Bak**	sgBak.2	CTCCTACAGCACCATGGGGC	3
**Bax**	sgBax.2	CCATTCGCCCTGCTCGATCC	3
**Caspase-1**	sgCasp1.2	GACATTCCCTTCTGAGCCTG	4
**MLKL**	sgMLKL.3	GGAGCTCTCGCTGTTACTTC	2
**MLKL**	sgMLKL.6	TCATCTCTTCACACCGTTTG	2
**PARP1**	sgPARP1.1	TTCTAGTCGCCCATGTTTGA	2

### TA cloning

TA cloning kit (Life technologies, ref. no. K202020) was used according to manufacturer's instructions to sequence DNA fragment containing the region where Cas9 was guided by a sgRNA.

### Electron microscopy

NB1 cells were plated in poly-L-lysine (0.01%, Sigma-Aldrich, ref. no. P4832)-coated glass slides (LabTek Chamber Slides, ref. no. 177399) at a density of 300,000 cells per slide (area = 1.8 cm^2^), cultured for 24 hours. The Raji Burkitt lymphoma cells were cultured at a density of 200,000 cells per ml. The cells were treated as described in the figures. Cells were then fixed 2 hours in 2.5% glutaraldehyde (Electron Microscopy Sciences, ref. no. 16220) dissolved in 100 mM phosphate buffer (PB), pH7.4. After three washes in PB, cells were postfixed for 1 hour in 1% osmium tetroxide (Electron Microscopy Sciences, ref. no. 19150) in PB and then stained with ethanol 70% containing 1% uranyl acetate (Sigma-Aldrich, ref. no. 73943) for 20 min. Raji and NB1 cells were dehydrated in graded alcohol series and embedded in epon (Electron Microscopy Sciences, ref. no. 13940). Ultrathin sections (with silver to gray interference) were cut with a diamond knife (Diatome), mounted on Formvar-coated single slot grids, and then counterstained with 3% uranyl acetate for 10 min and then with lead citrate (0.2%, Sigma-Aldrich, ref. no. 15326) for 10 min. Sections were visualized using a Philips CM100 transmission electron microscope.

## SUPPLEMENTARY FIGURES


