# Supplementary material for: The TAT-RasGAP317-326 anti-cancer peptide can kill in a caspase-, apoptosis-, and necroptosis-independent manner
Source: Oncotarget. 2016 Sep 2;7(39):64342–59. doi: 10.18632/oncotarget.11841 (PMC5325447; doi:10.18632/oncotarget.11841)
Supplement: Supplementary file 1 [file oncotarget-07-64342-s001.pdf]

# The TAT-RasGAP$_{317-326}$ anti-cancer peptide can kill in a caspase-, apoptosis-, and necroptosis-independent manner

## SUPPLEMENTARY FIGURES

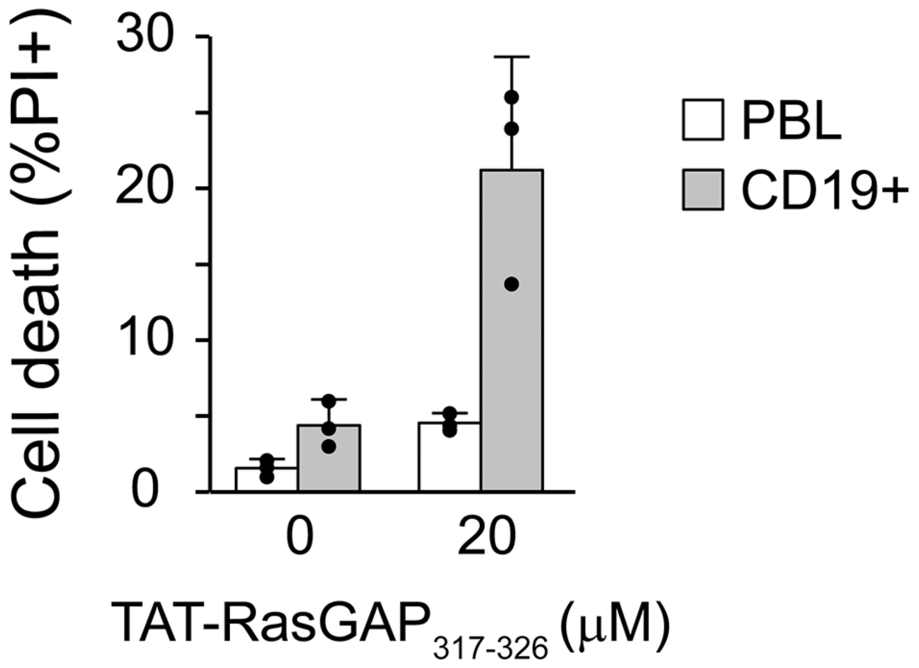


**Supplementary Figure S1: Effect of TAT-RasGAP$_{317-326}$ on the survival of PBLs and B cells.** PBLs from 3 different healthy donors were left untreated or treated with 20 µM TAT-RasGAP$_{317–326}$ for 16 hours. Then, cells were washed twice with PBS and B cells were stained with FITC-labelled anti-CD19 antibody. Cell death was assessed by flow cytometry after PI staining.

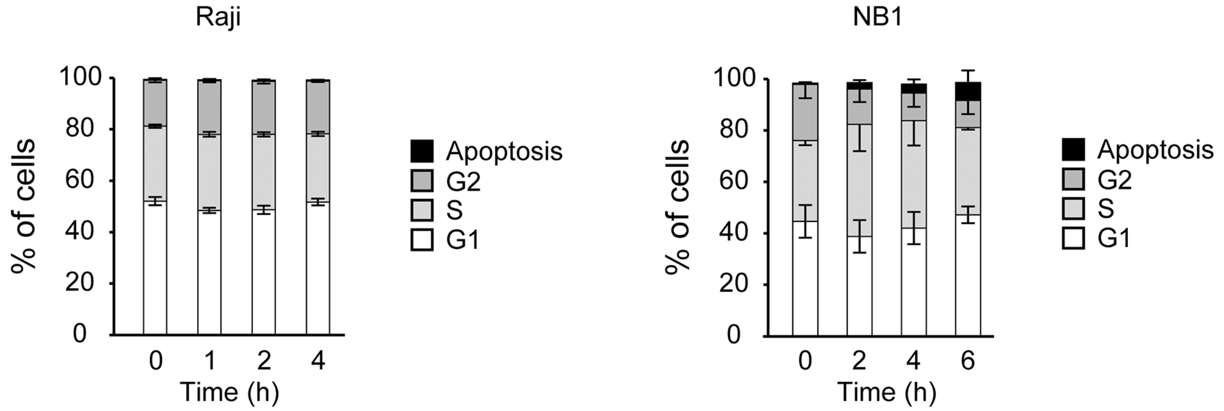


**Supplementary Figure S2: Effect of TAT-RasGAP$_{317-326}$ on cell cycle.** Raji cells and NB1 cells were treated for the indicated periods of time with 20 and 40 µM TAT-RasGAP$_{317-326}$, respectively. Cells were collected and cell cycle analyzed by flow cytometry. Results correspond to the mean +/- 95% CI of 3 independent experiments.

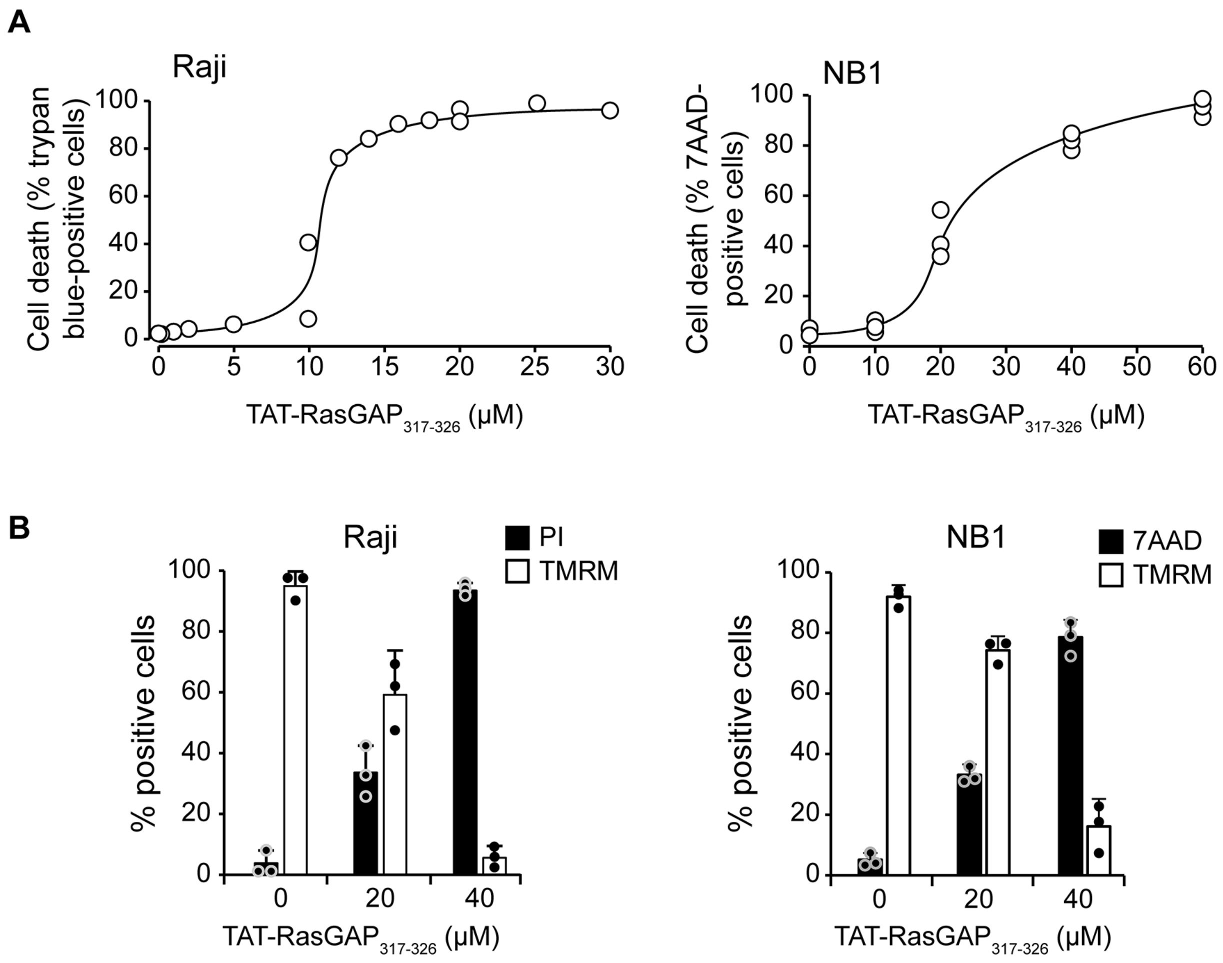


**Supplementary Figure S3: Sensitivity of Raji and NB1 cells to TAT-RasGAP$_{317-326}$-mediated death and effect of the peptide on mitochondrial potential. A.** Raji cells were treated with different concentrations of TAT-RasGAP$_{317-326}$ for 72 hours and cell death was determined by trypan blue exclusion. NB1 cells were treated with different concentrations TAT-RasGAP$_{317-326}$ for 24 hours and cell death was determined by flow cytometry using 7AAD. **B.** Raji and NB1 cells were treated with 0, 20 and 40 µM TAT-RasGAP$_{317-326}$ for 16 and 24 hours, respectively. Cell death and mitochondrial membrane potential were then analyzed by PI and TMRM staining using flow cytometry. Results correspond to the mean +/- 95% CI of 3 independent experiments.

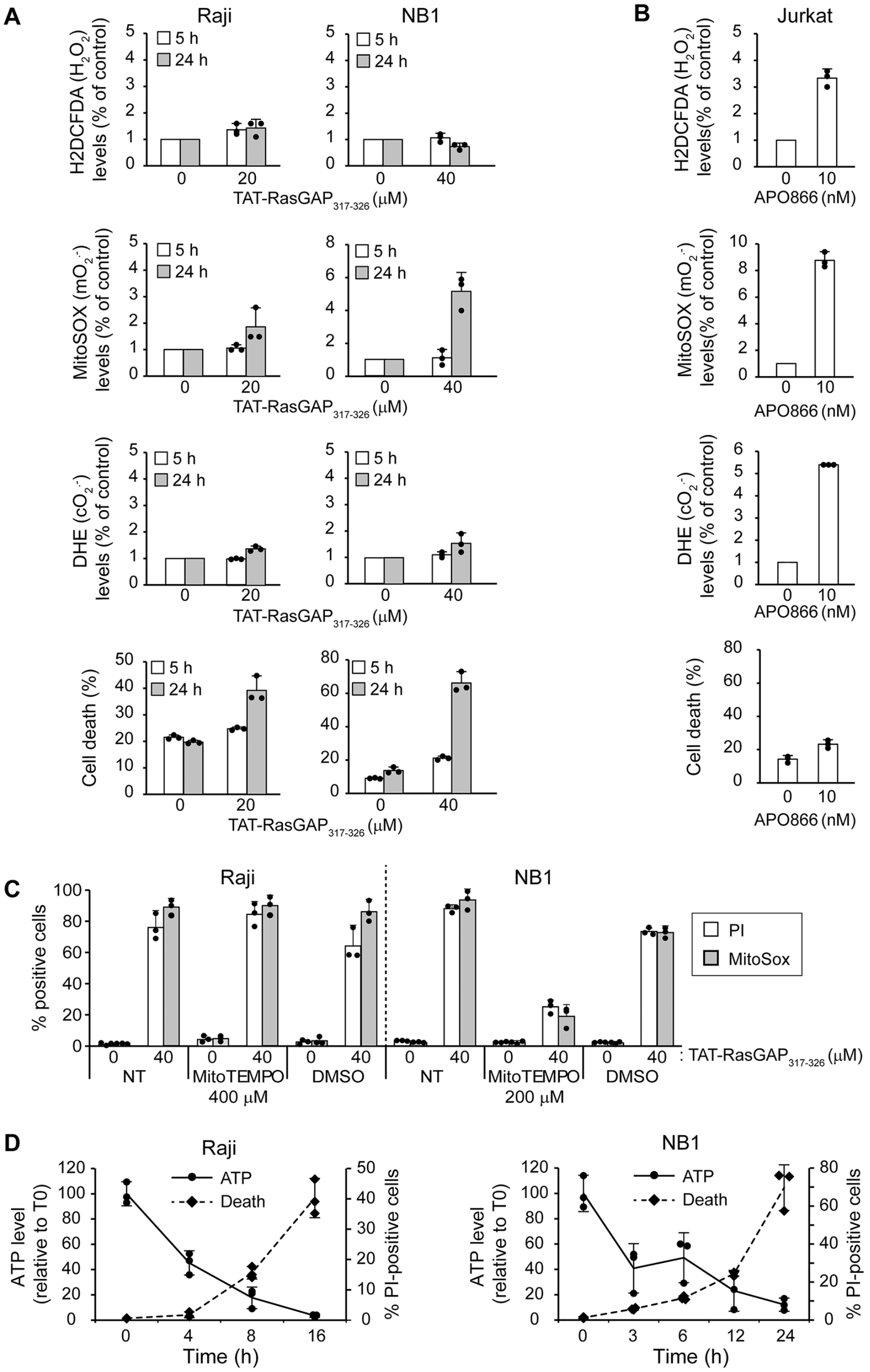


**Supplementary Figure S4: Effect of TAT-RasGAP$_{317-326}$ on ROS production. A.** Intracellular ROS production was detected in Raji or NB1 cells after treatment with 20 and 40 μM TAT-RasGAP$_{317-326}$, respectively. Cell death (corresponding to the % of Annexin-V and/or 7AAD-positive cells) was determined by flow cytometry. $H_2O_2$, $cO_2^{\cdot-}$ and $mO_2^{\cdot-}$ superoxides were detected for the indicated periods of time by flow cytometry staining using carboxy-H2DCFDA, DHE and MitoSOX fluorescent probe, respectively. **B.** Jurkat cells were treated for 48 hours with 10 nM APO866 (a potent ROS inducing agent). Cell death and ROS production were determined by flow cytometry using specific probes as described above. **C.** Cells were pretreated for 2 hours with or without the indicated concentrations of MitoTEMPO and then treated or not with 40 μM TAT-RasGAP$_{317-326}$ for 24 hours. Cell death and $mO_2^{\cdot-}$ were measured by flow cytometry using PI and MitoSox, respectively. The results are expressed as percentage of cells that are PI-positive (PI) or MitoSox-positive. **D.** Intracellular ATP levels were determined at the indicated periods of time for Raji and NB1 cells treated with 20 and 40 μM TAT-RasGAP$_{317-326}$, respectively. Each sample was normalized to protein content and to the mean basal ATP level (T0). Cell death was assessed by flow cytometry after PI staining. Results correspond to the mean +/- 95% CI of 3 independent experiments.

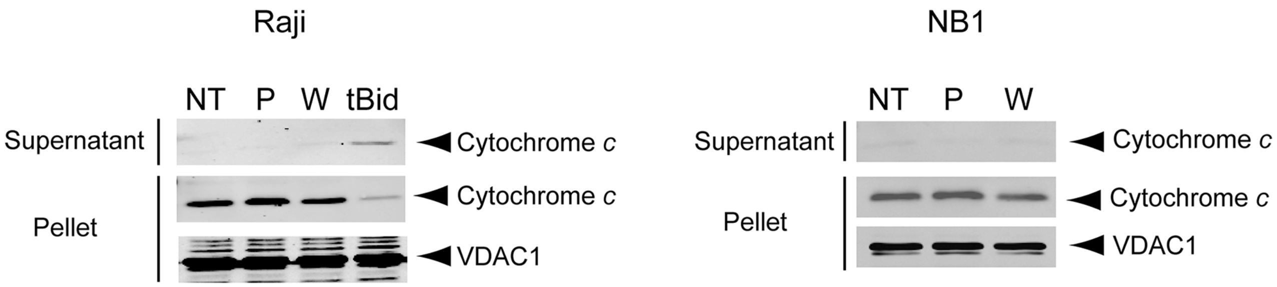


**Supplementary Figure S5: Effect of TAT-RasGAP$_{317\text{-}326}$ on isolated mitochondria.** Isolated mitochondria were left untreated or treated with 20 µM TAT-RasGAP$_{317–326}$ (P), 20 µM TAT-RasGAP$_{317–326}$ (W317A) (W) or 40 nM tBid for 30 min at 37°C. After centrifugation, supernatant and pellet were analyzed by Western blotting.

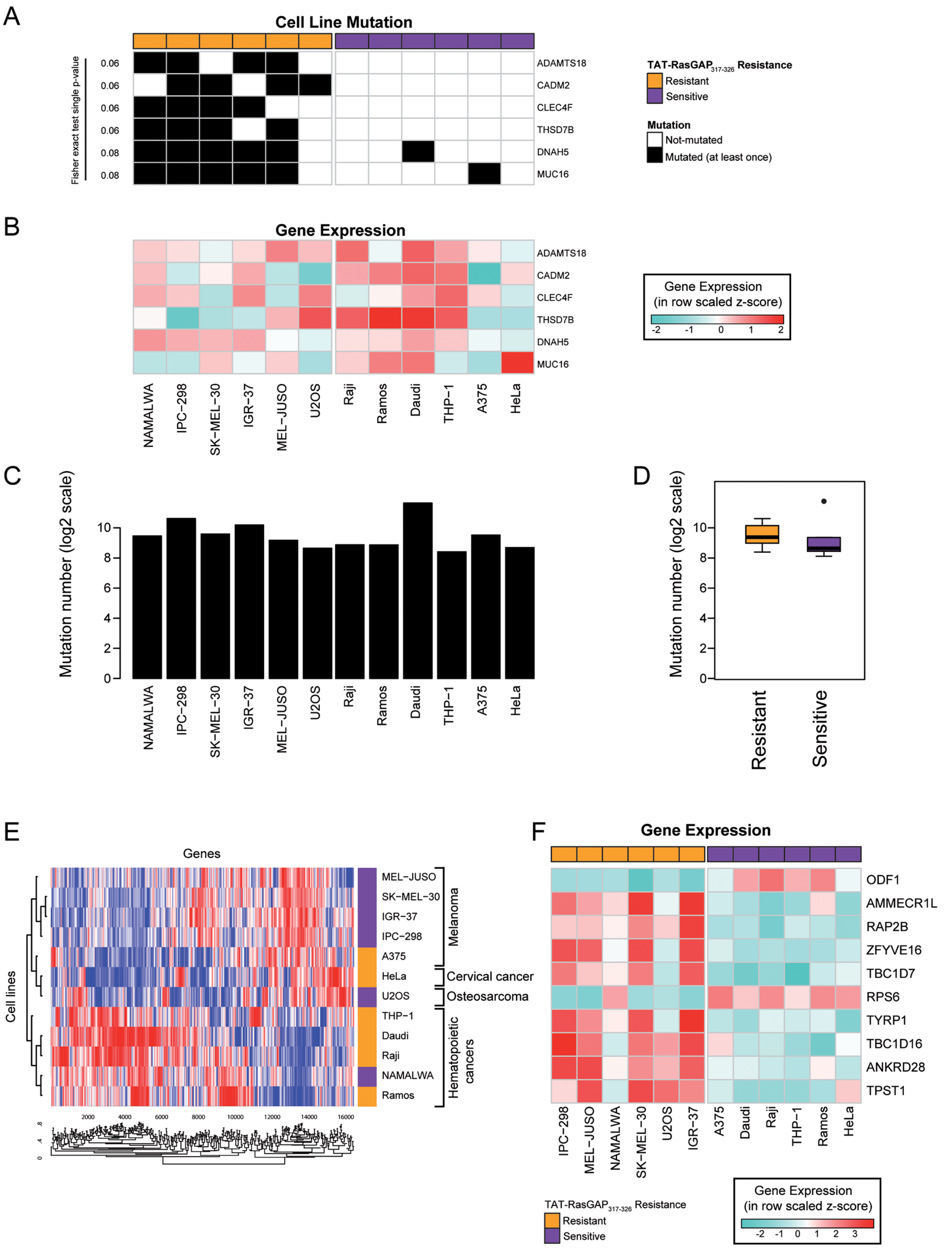


**Supplementary Figure S6: Comparison of mutational count and gene expression profiles between TAT-RasGAP$_{317-326}$-sensitive and -resistant cell lines. A.** Assessment of mutational enrichment according to TAT-RasGAP$_{317-326}$ sensitivity with black representing mutated genes and white denoting no mutation. The six most highly enriched genes are displayed. **B.** Heatmap representing the expression of the six most mutated genes. **C.** Number of non-synonymous somatic mutations among the twelve cell lines. **D.** Distribution of somatic mutation number in resistant and sensitive cell lines. **E.** Heatmap representing gene expression of the twelve different cell lines, with a gradient from blue (low expression) to red (high expression). **F.** Heatmap displaying the ten most differentially expressed genes between resistant and sensitive cell lines.
